# Fabrication of Er^3+^/Yb^3+^ Co-Doped Bi_5_O_7_I Microsphere With Upconversion Luminescence and Enhanced Photocatalytic Activity for Bisphenol A Degradation

**DOI:** 10.3389/fchem.2020.00773

**Published:** 2020-09-03

**Authors:** Baowei Cao, Siwen Gong, Siyaka Mj Zubairu, Lingna Liu, Yunhua Xu, Lei Guo, Rui Dang, Gangqiang Zhu

**Affiliations:** ^1^School of Chemistry and Chemical Engineering, Yulin University, Yulin, China; ^2^School of Physics and Information Technology, Shaanxi Normal University, Xi'an, China; ^3^Department of Chemistry, Federal University Gashua, Gashua, Nigeria

**Keywords:** doping, semiconductor, microsphere, upconversion, heterojunction photocatalytic activity, NO removal, Rhodamine B

## Abstract

Er^3+^/Yb^3+^ co-doped Bi_5_O_7_I uniform porous microsphere photocatalysts were synthesized by a two-step chemical method, which possesses excellent photocatalytic performance and upconversion luminescence property. The photocatalytic performance of the photocatalysts was studied by degradation of bisphenol A in aqueous solution under visible light and different monochromatic light irradiation. The photocatalytic performance of Er^3+^/Yb^3+^ co-doped Bi_5_O_7_I sample is better than that of the pristine Bi_5_O_7_I and Er^3+^-doped Bi_5_O_7_I samples. Moreover, Er^3+^/Yb^3+^ co-doped Bi_5_O_7_I possesses photocatalytic ability with a red light monochromatic LED lamp (3 W, λ = 630 nm) and an infrared monochromatic LED lamp (100 W, λ = 940 nm) irradiation whose wavelength is longer than the absorption-limiting wavelength of pristine Bi_5_O_7_I sample. This phenomenon further verified that the upconversion property of Er^3+^ and Yb^3+^ causes the improved photocatalytic efficiency of Er^3+^/Yb^3+^ co-doped Bi_5_O_7_I sample.

**Graphical Abstract:**
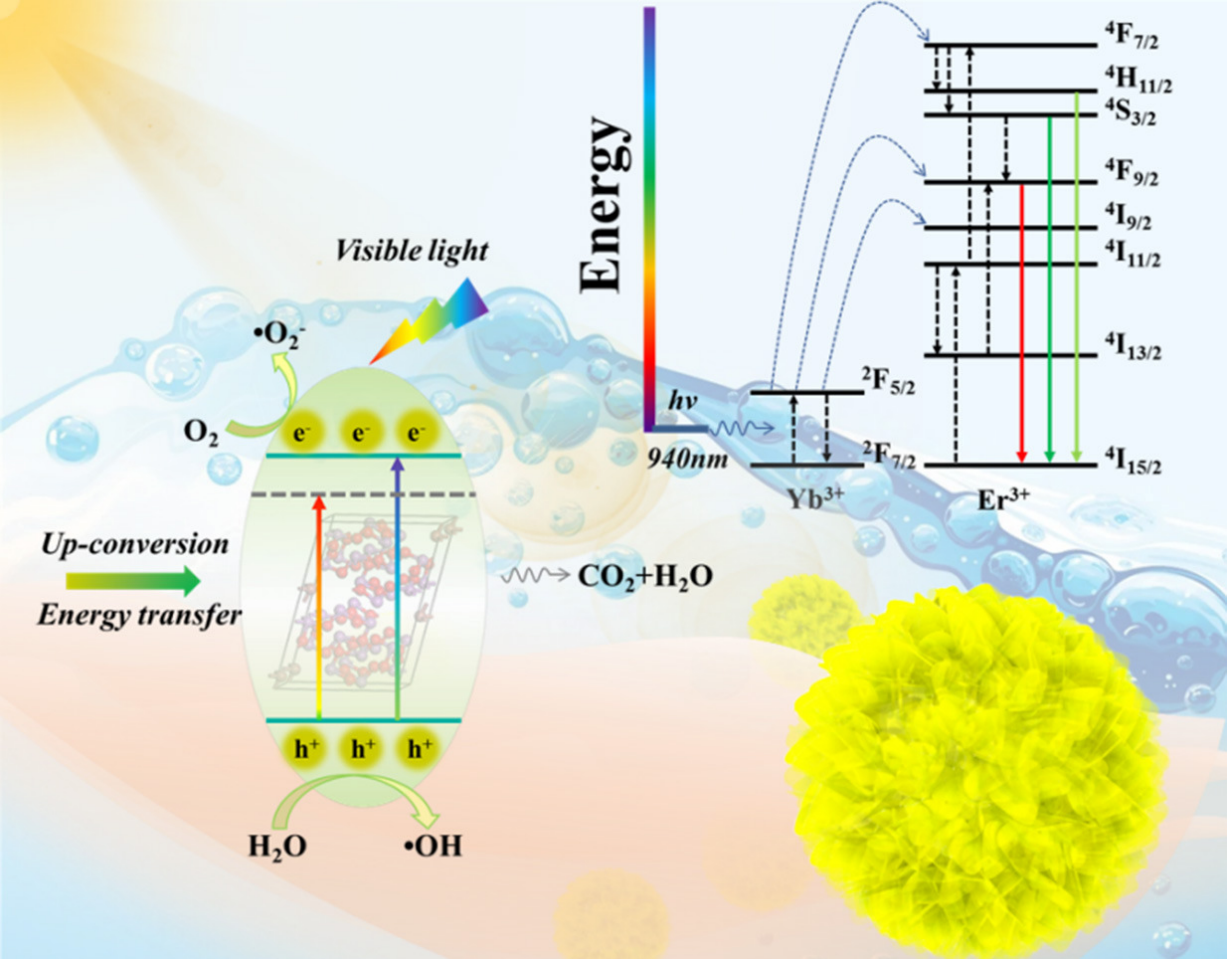
Er^3+^/Yb^3+^ co-doped Bi_5_O_7_I porous microsphere photocatalysts show efficiency photocatalytic properties for bisphenol A degradation under visible and near-infrared light irradiation.

## Introduction

Upconversion is a particular type of photoluminescence (PL), which converts low-energy excitation light into high-energy emission light through a multiphoton absorption process (Obregón and Colón, 2014a; Chuai et al., 2015; Ma et al., 2015; Fu et al., 2017). For this excellent characteristic, many upconverting materials, such as YF_3_ and NaYF_4_, have been used as powerful assistants to combine with semiconductor photocatalysts to improve light utilization recently (Huang et al., 2012; Li et al., 2013). For instance, Qin et al. (2010) reported that the graphene-supported NaYF_4_:Yb^3+^, Tm^3+^, and N-doped P25 nanocomposite photocatalysts exhibit outstanding photocatalytic efficiency, because upconverting materials can effectively convert long-wavelength infrared (IR) light into short-wavelength light (such as visible light). The semiconductors in the composite photocatalysts can absorb the converted short-wavelength light to make full use of incident light. However, many up-conversion materials did not have photocatalytic performance because of their large band gap (Wang et al., 2013; Xu et al., 2013). Therefore, it is important to fabricate single-phase photocatalyst with excellent photocatalytic activity and upconversion property.

Bi_5_O_7_I as a novel semiconductor photocatalytic material with an optical band gap of ~2.8 eV has received a lot of attention (Zhang et al., 2020a). The lamellar crystallographic structure of Bi_5_O_7_I can form an internal electrostatic field whose direction is vertical to the atom layer. The internal electrostatic field can promote the separation of photo-generated electron-hole pairs (Lan et al., 2020). However, the shortcomings of low light absorption and transmission efficiency of carriers still limit its photocatalytic activity. It is well-known that combining Bi_5_O_7_I with other semiconductors to form heterojunction could improve the separation rate of photo-generated charge carriers and show enhanced photocatalytic efficiency for pollutants degradation (Liu et al., 2015; Zhang et al., 2020b). In addition, our previous report indicated that the doping of Er^3+^ into the Bi_5_O_7_I can broaden the photo-response range due to the upconversion effect (Hojamberdiev et al., 2020), but the light conversion is not thorough enough. It can be inferred that the Er^3+^/Yb^3+^ co-doping would cause more intensive upconversion fluorescence effect (Ding et al., 2016), which can enhance photocatalytic degradation properties for pollutions with full spectral solar light response.

In this work, uniform Er^3+^/Yb^3+^ co-doped Bi_5_O_7_I microsphere photocatalysts were prepared by a two-step hydrothermal and thermal–decomposition method. The as-prepared photocatalysts have excellent photocatalytic performance and upconversion luminescence property. From the results of photocatalytic performance tests under the illumination of visible and monochromatic light and trapping experiments, the detailed mechanism of improved photocatalytic activity was also proposed.

## Experimental

The synthesis methods of Bi_5_O_7_I and 6%Er^3+^-doped Bi_5_O_7_I samples are detailed in the Supporting Information and the prepared samples were recorded as BOI and 6EBOI, respectively. In addition, the synthesis method of Yb^3+^/Er^3+^ co-doped Bi_5_O_7_I samples was similar to that previously reported (Zhang et al., 2019), except the addition of 2 to 18% Yb(NO_3_)_3_ 6H_2_O. The prepared samples were recorded as 2Y6EBOI, 4Y6EBOI, 6Y6EBOI, 12Y6EBOI, and 18YEBOI, respectively. The characterization and photocatalytic test are also described in the Supporting Information.

## Results and Discussion

### XRD Analysis

**Figure 1:**
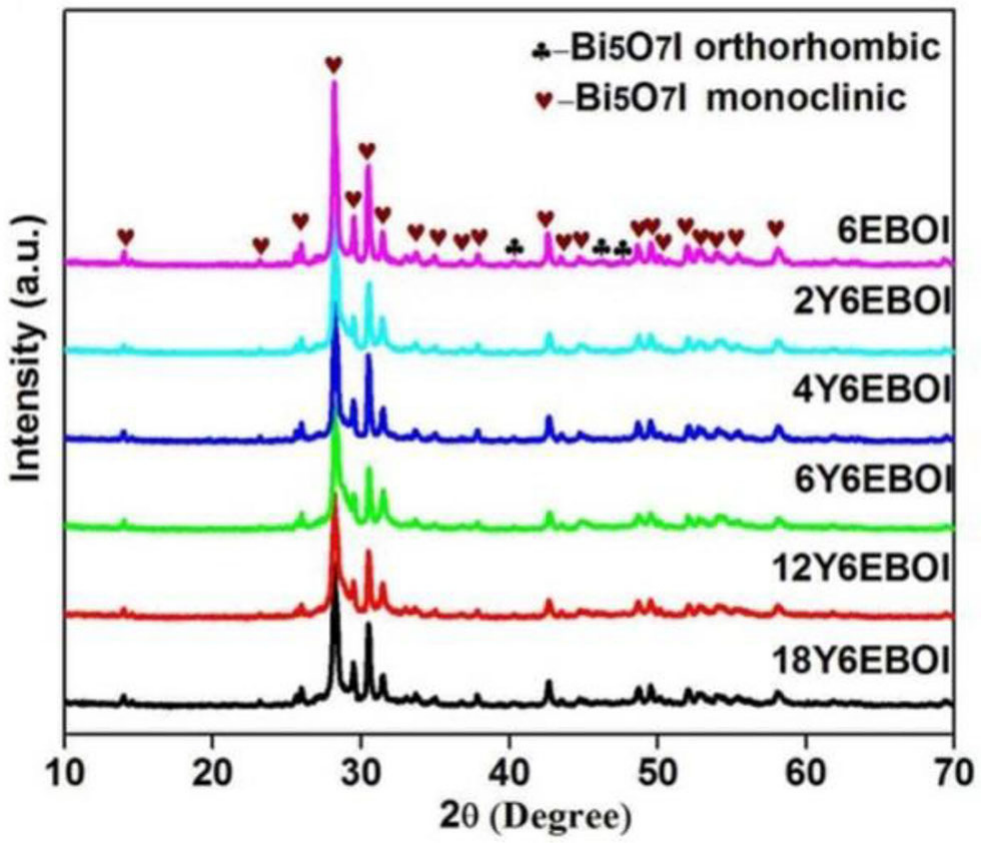
XRD patterns of 6EBOI, 2Y6EBOI, 4Y6EBOI, 6Y6EBOI, 12Y6EBOI, and 18Y6EBOI samples.

The XRD patterns of 6EBOI and YEBOI samples are shown in Figure 1, and all the prepared samples are crystallized well. As previously reported, the Bi_5_O_7_I synthesized without any doping corresponds with orthorhombic phase Bi_5_O_7_I (JCPDS 40-0548) (Hojamberdiev et al., 2020). However, the synthesized 6EBOI sample is in accordance with orthorhombic phase Bi_5_O_7_I and monoclinic phase (JCPDS 38-0669) Bi_5_O_7_I. In addition, the phase structure of the samples is completely converted into monoclinic Bi_5_O_7_I after doping with Yb^3+^. Moreover, as the Yb^3+^ doping content increases, the width of these peaks broadens and the intensity decreases, especially the (004) peak. This is because the doping of Yb^3+^ and Er^3+^ limits the growth of Bi_5_O_7_I crystals. According to the previous reports, the existence of Yb^3+^ and Er^3+^ in the compound can cause phase transition from the orthorhombic phase to the monoclinic phase (Lin et al., 2014; Obregón and Colón, 2014b; Obregón et al., 2014).

### Scanning Electron Microscope Analysis

**Figure 2:**
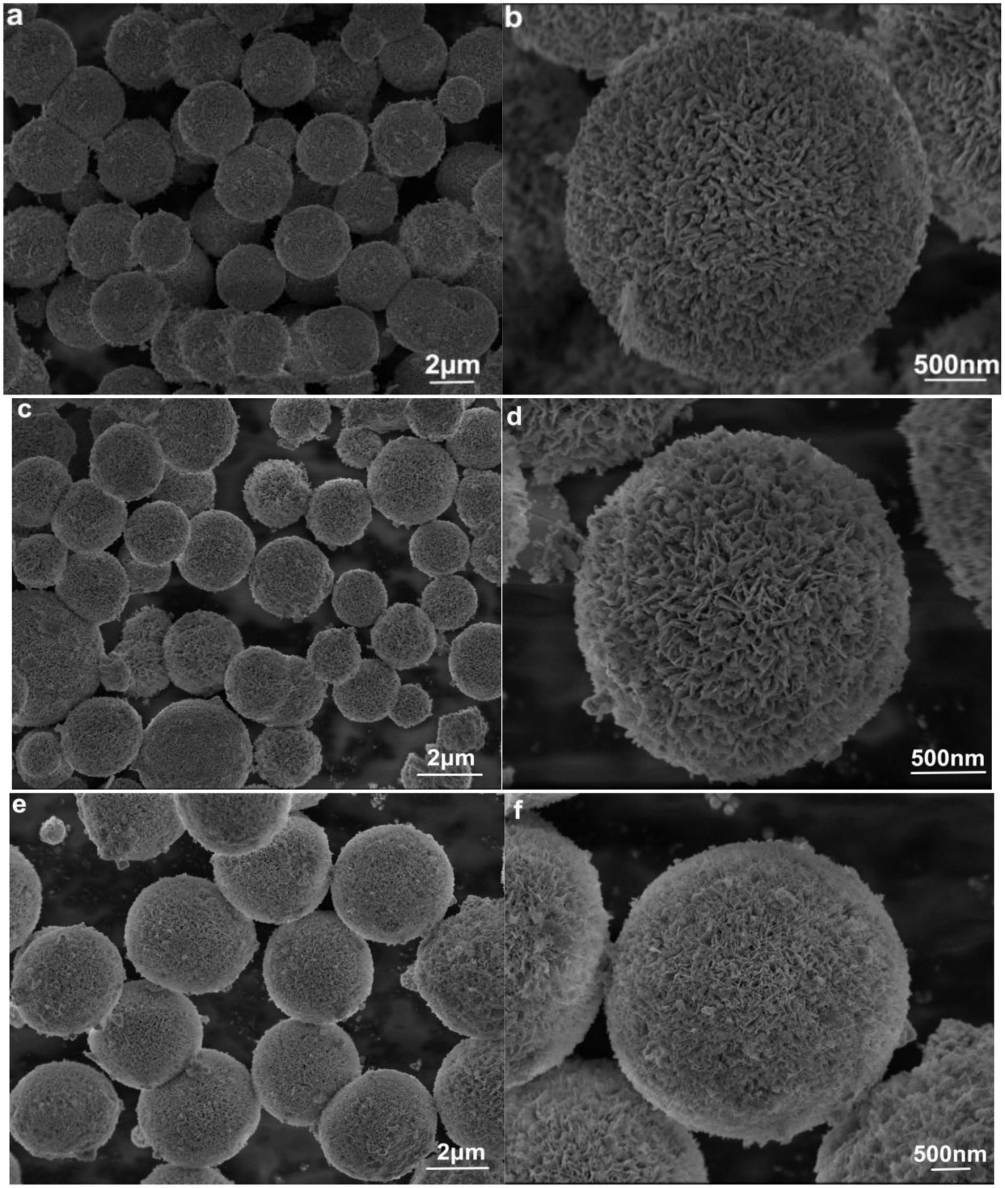
SEM images of BOI **(a,b)**, 6EBOI **(c,d)**, and 6Y6EBOI **(e,f)** samples.

**Figure 3:**
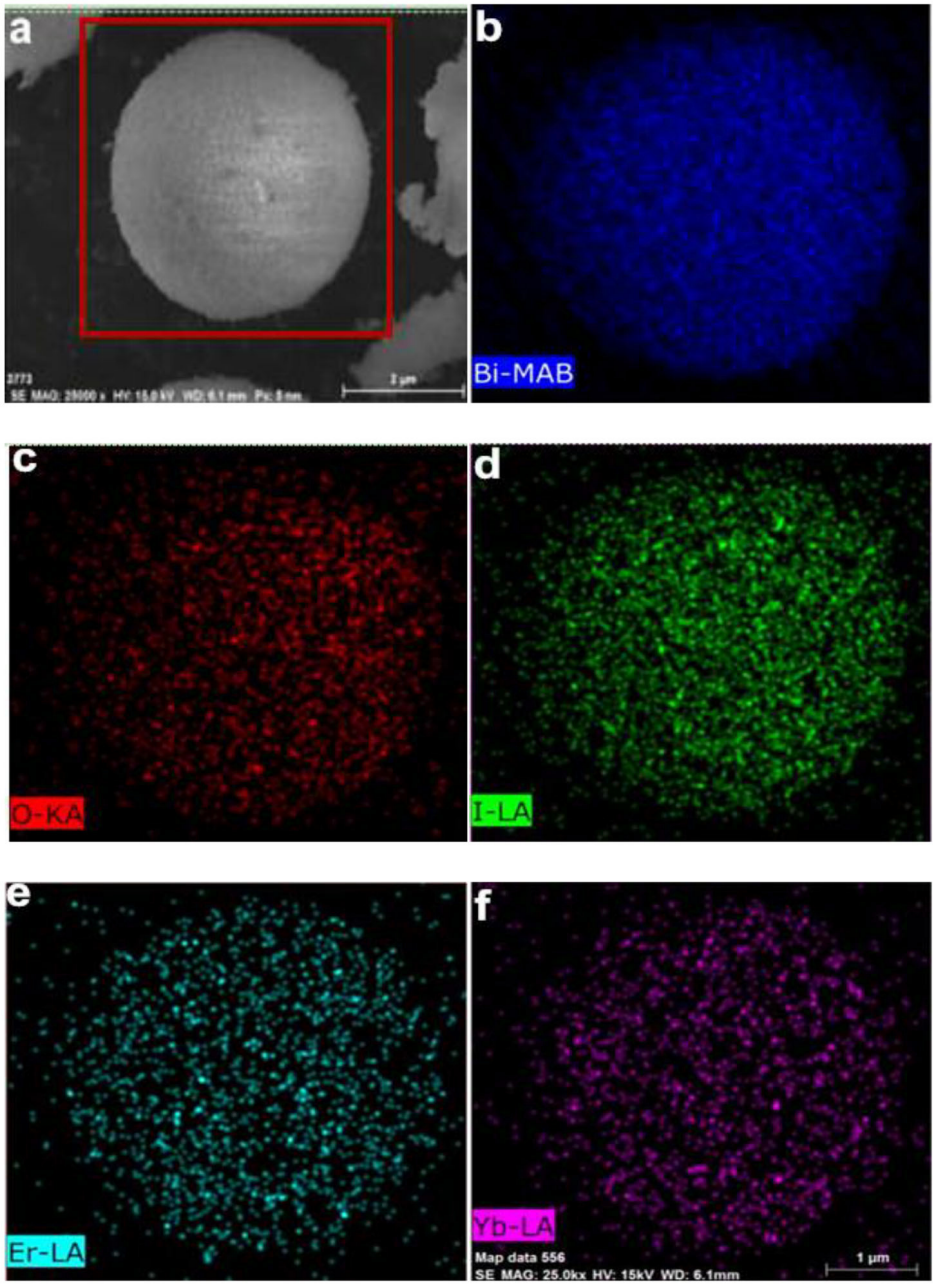
SEM image **(a)**, Bi **(b)**, O **(c)**, I **(d)**, Er **(e)**, and Yb **(f)** EDS mapping images of 6Y6EBOI sample.

Figure 2 displays the scanning electron microscope (SEM) images of the synthesized pure BOI, 6EBOI, and YEBOI samples. As shown in Figure 2a, the BOI sample has a uniform porous spherical morphology with a radius in the range of 1 to 1.5 μm. From Figure 2b, the high-resolution SEM image shows these spheres are stacked by numerous nanosheets. While Figures 2c–f show the SEM images of 6EBOI and 6Y6EBOI samples, respectively. The Yb^3+^/Er^3+^ doping has little effect on the morphology, and all the as-prepared samples also have the uniform porous spherical morphology. Energy dispersive spectrometer (EDS) mapping was performed further to analyze the elemental distribution in the 6Y6EBOI sample (Figure 3). It is observed that the Bi, O, I, Er, and Yb elements are well distributed over the whole microsphere.

### XPS Analysis

**Figure 4:**
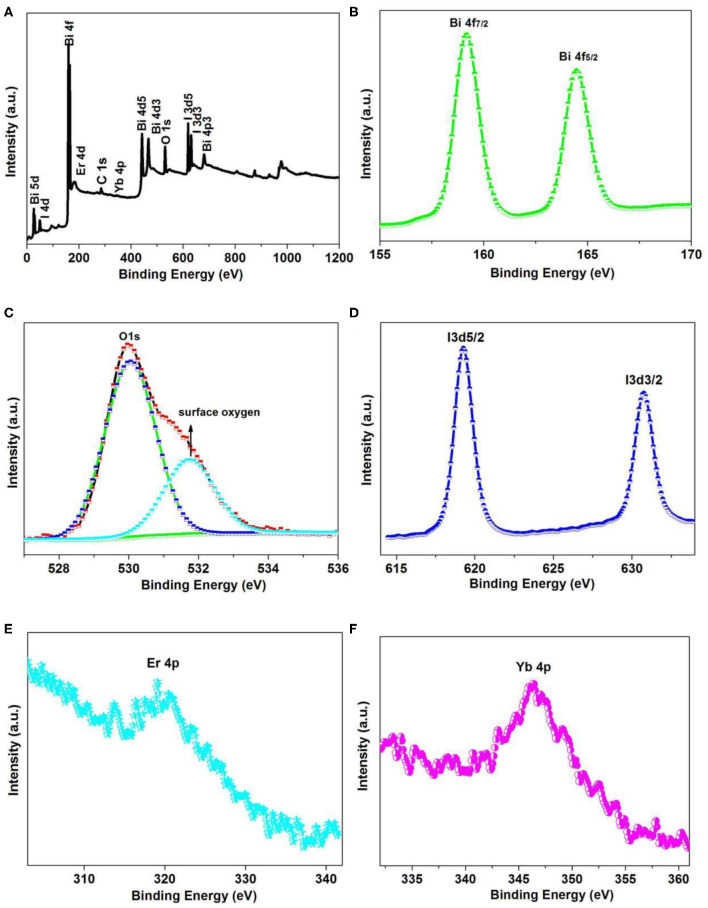
Survey XPS spectra **(A)**, high-resolution XPS spectra of **(B)** Bi 4f, **(C)** O 1s, **(D)** I 3d, **(E)** Er 4p, and **(F)** Yb 4p of the 6Y6EBOI sample.

In order to investigate the elemental composition, XPS analysis was performed on the 6Y6EBOI sample, and the consequences are presented in Figure 4. The survey spectrum in Figure 4A clearly reveals the compound consists of Bi, O, I, Er, and Yb elements. There are two peaks at ~164.4 and 158.9 eV (Figure 4B), which are ascribed to Bi 4f_7/2_ and Bi 4f_5/2_ (Liu et al., 2017), respectively. In Figure 4C, it is observed that the O 1s peak is located at 529.5 and 531.4 eV, which corresponds to the lattice oxygen and surface-adsorbed oxygen in the prepared sample (Zhu et al., 2019). The peaks located at 619.4 and 630.6 eV (Figure 4D) correspond with the I 3d_5/2_ and I 3d_3/2_ (Rao et al., 2019). It is also seen that the Er 4p (Figure 4E) and Yb 4p (Figure 4F) peaks are located at 321.1 and 346.5 eV, which corresponds with the Er^3+^ and Yb^3+^ (Hou et al., 2012; Reszczynska et al., 2015), respectively. Thus, the XPS results indicate that the Er^3+^ and Yb^3+^ were triumphantly doped into the Bi_5_O_7_I sample.

### Ultraviolet-vis DRS Analysis

**Figure 5:**
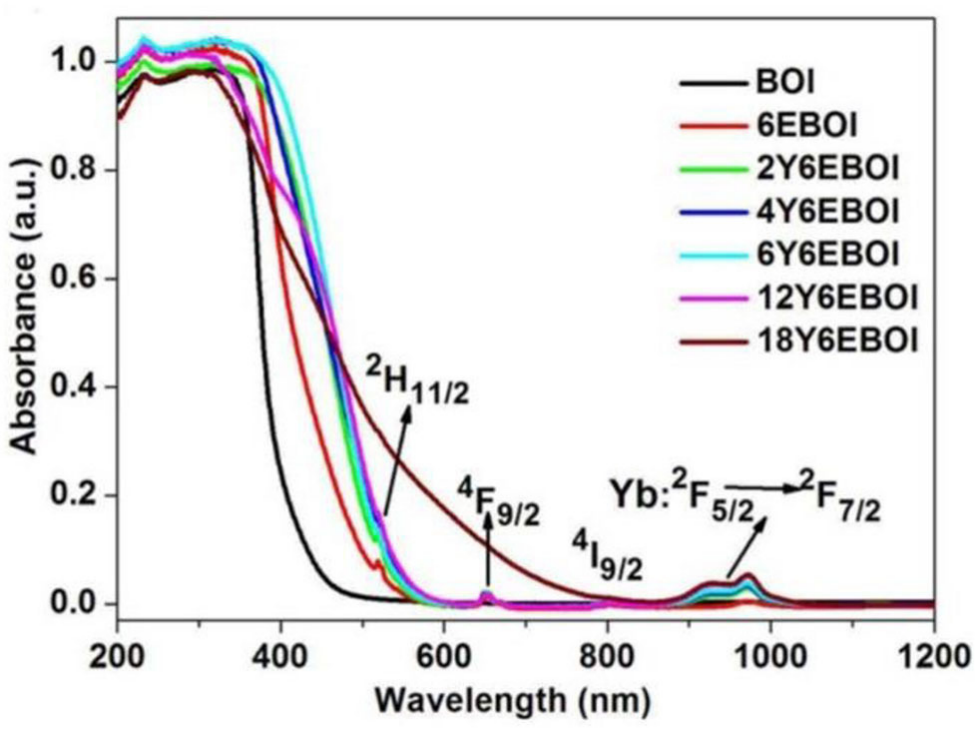
UV-vis adsorption spectra of synthesized pure BOI, 6E BOI, and Yb^3+^/6%Er^3+^ co-doped Bi_5_O_7_I photocatalysts.

The ultraviolet–visible (UV-vis) absorption spectra of the prepared samples are depicted in Figure 5. The adsorption edge of pure BOI is shorter than 450 nm, indicating that pristine BOI could be excited by the ultraviolet light and a small fraction of visible light. Compared with pure BOI, the visible light absorption of Yb^3+^/Er^3+^-doped BOI samples undergoes a significant redshift. It can be seen that three peaks are located at 522, 655, and 797 nm for 6EBOI. This is attributed to the upconversion effect from the ^4^I_15/2_ ground state to ^2^H_2/11_, ^4^F_9/2_, and ^4^I_9/2_ states of Er^3+^ (Rodríguez et al., 2013; Xu et al., 2014). An exception absorption peak at nearly 950 nm for the Yb^3+^-doped 6EBOI sample is also clearly observed, which is attributed to the upconversion conversion from the ^2^F_5/2_ ground state to ^2^F_7/2_ states of Yb^3+^ (Wang et al., 2014).

**Figure 6:**
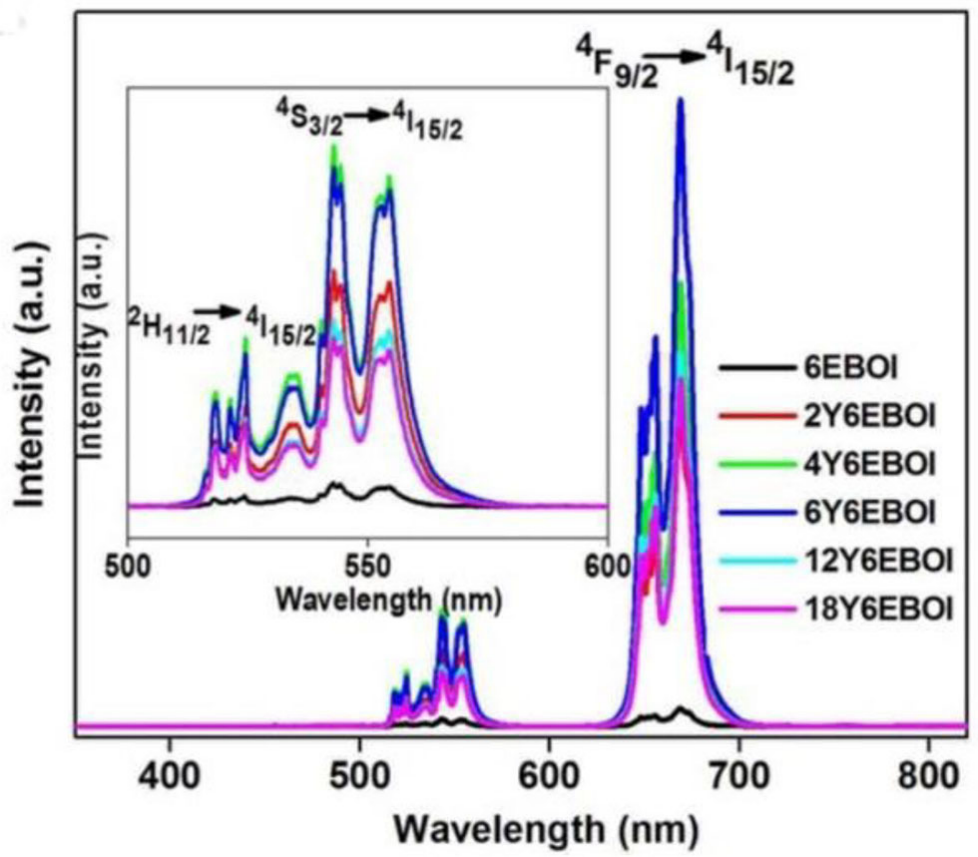
Upconversion spectra of Er^3+^-doped Bi_5_O_7_I; and Yb^3+^-Er^3+^co-doped Bi_5_O_7_I sample under 980-nm laser excitation.

**Figure 7:**
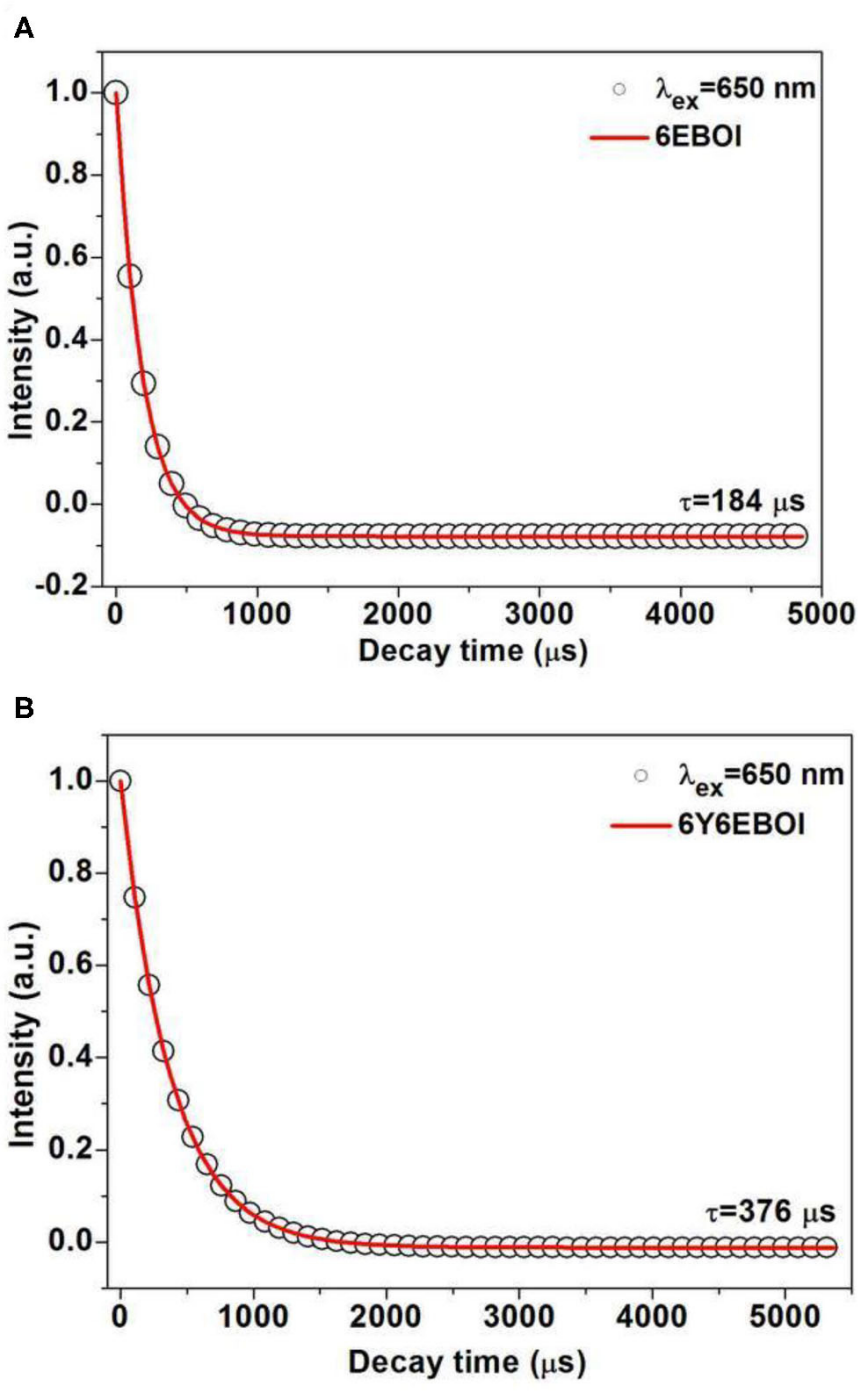
Decay curves of ^4^F_9/2_-^4^I_15/2_ transitions of Er^3+^ ions for the corresponding 6EBOI **(A)** and 6Y6EBOI **(B)** samples.

In order to know the cause of these new peaks in the visible and near IR (NIR) light range, the upconversion spectra of YEBOI samples were carried out. Figure 6 exhibits the UC emission spectra (350–800 nm) of YEBOI samples. It shows that there are two green emission bands near 533 and 547 nm, and a red emission band near 654 nm after excitation by an NIR laser (λ = 980 nm). The former between 515 to 538 nm and 540 to 560 nm are ascribed to the ${}^{2}{\text{H}}_{11/2}\to^{4}{\text{I}}_{15/2}$ and ${}^{4}{\text{S}}_{3/2}\to^{4}{\text{I}}_{15/2}$ transitions (Zhang et al., 2005; Sun et al., 2011; Mahalingam et al., 2013). The latter between 640 and 680 nm corresponds with the transition of ^4^F_9/2_ to ^4^I_15/2_. It is very clear that the intensity of the green and red emission bands increases over Yb^3+^-doped 6EBOI sample. Therefore, the observation results indicate that the new absorption bands appearing in the UV-vis DRS spectra are caused by the upconversion radiation of the YEBOI system (Liu et al., 2013; Bai et al., 2014; Zhou et al., 2015). It is well-known that the lifetime of the upconversion materials exhibits a positive correlation with the upconversion quantum yield (Dai et al., 2013). Thus, the luminescence decay curves of the as-synthesized 6EBOI and 6Y6EBOI are also compared under the excitation light with 650 nm wavelength (Figure 7). The decay curves of 6EBOI and 6Y6EBOI are 184 and 376 μs, respectively. Hence, the lifetime is significantly prolonged after Yb^3+^dopping compared with the 6EBOI sample. It is concluded that the tendency of lifetime variation is consistent with that of upconversion intensity variation.

### Photo-Degradation of Bisphenol A

**Figure 8:**
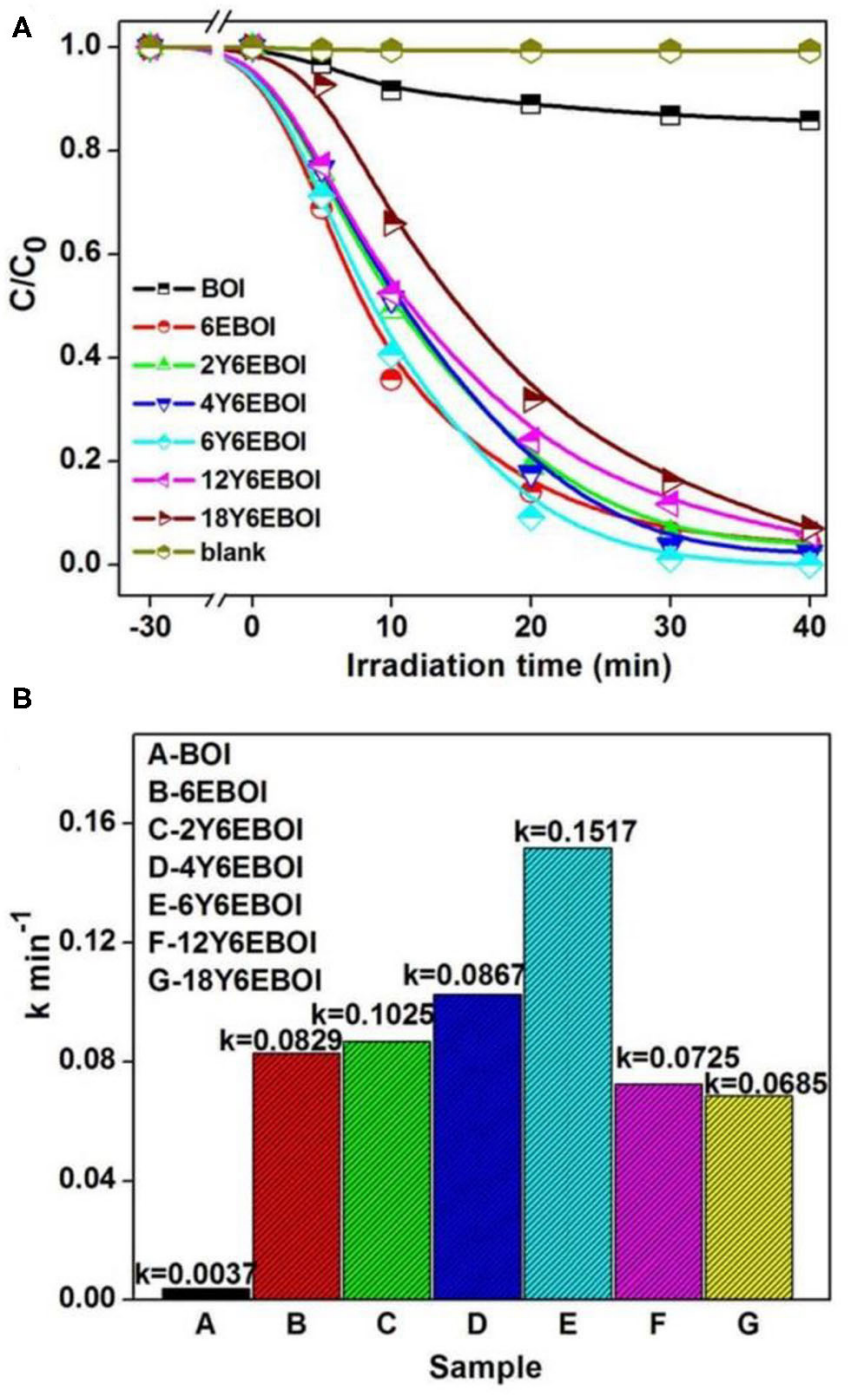
Variation of BPA concentration (*C*/*C*_0_) against photodegradation time **(A)** and photocatalytic reaction rate constant *k* **(B)** of BOI, 6EBOI and Yb^3+^/Er^3+^ co-doped Bi_5_O_7_I samples.

Bisphenol A (BPA) in aqueous solution is selected as target to be degraded, and the photocatalytic efficiency of the photocatalysts under visible light irradiation is shown in Figure 8. As indicated in Figure 8A, after visible light irradiation for 40 min, the photocatalytic rates of BOI, 6EBOI, 2Y6EBOI, 4Y6EBOI, 6Y6EBOI, 12Y6EBOI, and 18Y6EBOI are 14.1, 95.7, 95.9, 97.7, 100, 94.1, and 92.9, respectively. Therefore, the 6Y6EBOI sample shows the best photocatalytic performance of all the as-prepared samples in this work. According to the Langmuir–Hinshelwood kinetics model (Chen et al., 2012), the below formula is used to express the degradation process:

(1) $$\begin{array}{r} \text{ln }\left(C_{1}/C\right)\;=\;\text{kt} \end{array}$$

where *C*_1_ represents the amount of target removal object after the equilibrium is reached between adsorption and desorption (*t* = 0), and *C* represents the real-time concentration of the degradation (t). As shown in Figure S1, the *k*'s for BOI, 6EBOI, 2Y6EBOI, 4Y6EBOI, 6Y6EBOI, 12Y6EBOI, and 18Y6EBOI samples were calculated as approximately 0.0037, 0.0829, 0.0867, 0.1025, 0.1517, 0.0725, and 0.0685 min^−1^ (Figure 8B), respectively. The kinetic results for pristine BOI, 6EBOI, and 6Y6EBOI samples prove the remarkable enhancement photocatalytic efficiency after Yb^3+^ and Er^3+^ doping into BOI photocatalysts.

**Figure 9:**
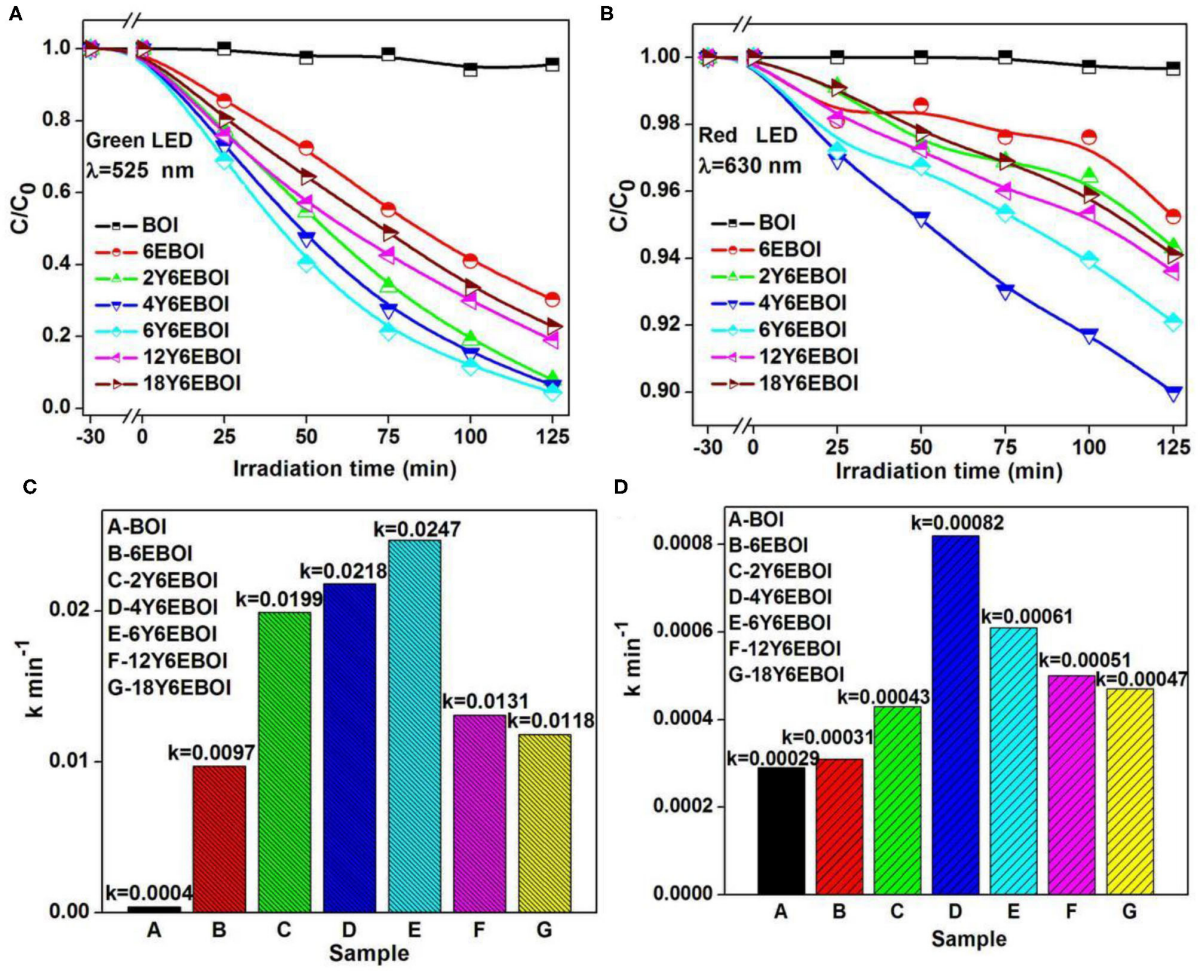
The photocatalytic activities of the Er^3+^-doped Bi_5_O_7_I **(A,C)** and Yb^3+^/Er^3+^-doped Bi_5_O_7_I **(B,D)** sample under different wavelength monochromatic light irradiation.

The photocatalytic activities of BOI, 6EBOI, 2Y6EBOI, 4Y6EBOI, 6Y6EBOI, 12Y6EBOI, and 18Y6EBOI samples under different wavelengths of monochromatic light were also studied. As shown in Figure 9, only 4 and 0.3% BPA was degraded under green (G) and red (R) light irradiation for 125 min over BOI, respectively. In particular, the degradation efficiencies of 6EBOI, 2Y6EBOI, 4Y6EBOI, 6Y6EBOI, 12Y6EBOI, and 18Y6EBOI samples are 69.8, 92.1, 93.6, 95.5, 81.1, and 77.3 (Figure 9A) under green light irradiation for 125 min, respectively. The degradation efficiencies of 6EBOI, 2Y6EBOI, 4Y6EBOI, 6Y6EBOI, 12Y6EBOI, and 18Y6EBOI samples are 4.8, 5.7, 10.1, 8.1, 6.4, and 5.9% under the red light irradiation for 125 min, respectively (Figure 9B). The *k*'s of BOI, 6EBOI, 2Y6EBOI, 4Y6EBOI 6Y6EBOI, 12Y6EBOI, and 18Y6EBOI samples calculated from the data were 0.0004, 0.0097, 0.0199, 0.0218, 0.0247, 0.0131, and 0.0188 min^−1^ in Figure 9C under green light irradiation, and 0.00029, 0.00031, 0.00043, 0.00082, 0.00061, 0.00050, and 0.00047 min^−1^ in Figure 9D under the illumination of red light, respectively. These results indicate that the 6Y6EBOI sample has the most excellent photocatalytic performance for BPA degradation than pure BOI, 6EBOI, and other Er^3+^/Yb^3+^ co-doped BOI samples.

**Figure 10:**
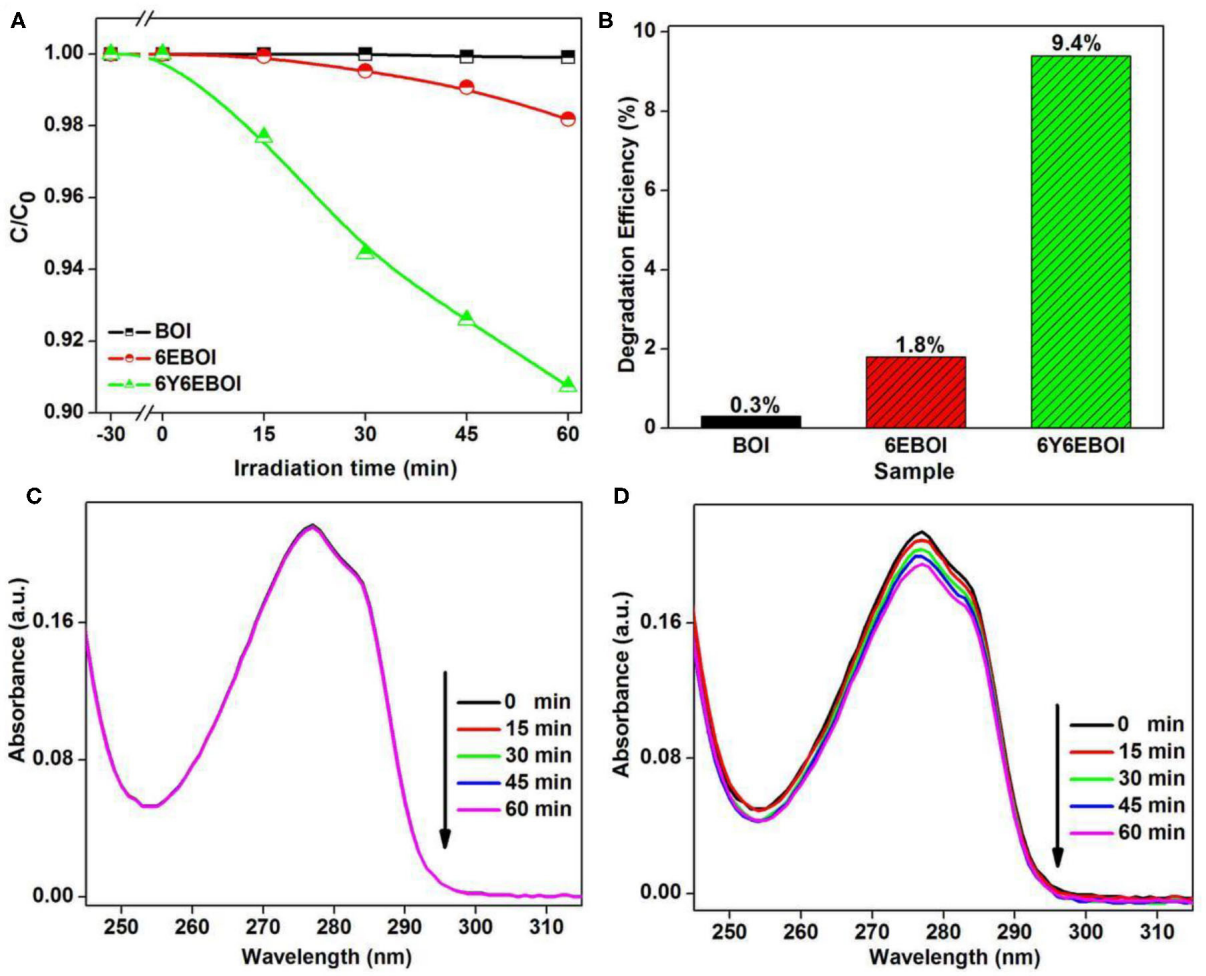
The photocatalytic activities **(A)** and degradation rate **(B)** of the BOI, 6EBOI, and 6Y6EBOI samples under 940-nm LED light irradiation, the variation of UV-vis spectral for the BPA in aqueous solution of BOI **(C)**, and 6Y6EBOI **(D)** samples.

Apparently, the photocatalytic activity was greatly improved after the doping of Yb^3+^ and Er^3+^ with Bi_5_O_7_I under visible light irradiation. It is more interesting that the 6Y6EBOI also possesses the best photocatalytic activity under NIR light (940 nm LED light) irradiation. For comparison, BOI and 6EBOI were also used as reference photocatalysts under the same experimental condition. As exhibited in Figures 10A,B, the photodegradation efficiencies of BOI, 6EBOI, and 6Y6EBOI samples are 0.3, 1.8, and 9.4%, respectively. The characteristic peak of BPA does not show any change even when the irradiation time reached 60 min over the BOI sample (Figure 10C). However, it has an obvious decrease of the peak intensity at 277 nm of BPA with the addition of 6E6YBOI sample as shown in Figure 10D. From the above photocatalytic results, the photocatalytic activity of the 6E6YBOI photocatalyst has excellent photocatalytic performance under visible light and NIR light irradiation.

### PL Spectra and *I-V* Analysis

**Figure 11:**
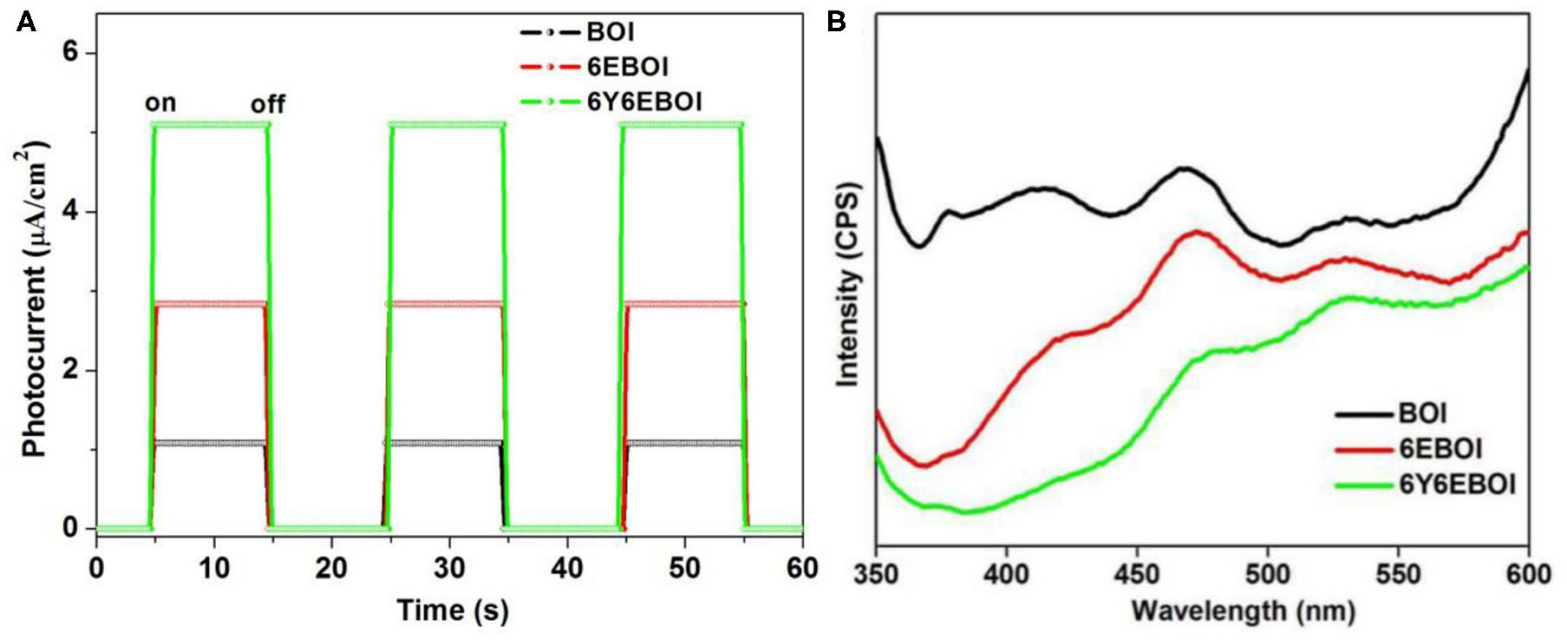
Transient photocurrent **(A)** and PL spectra **(B)** of the BOI, 6EBOI, and 6Y6EBOI samples.

The transient photocurrent (*I-V*) and PL are effective tests in displaying the separation ability of photo-generated carriers in photocatalytic research (Chang et al., 2019; Li et al., 2020a). The responses of *I-V* for BOI, 6EBOI, and 6Y6EBOI were also recorded under visible light irradiation. As shown in Figure 11A, the intensity of photocurrent signal of 6E6FBOI is much stronger than the pristine BOI and 6EBOI, which suggests the best excellent effective transfer ability of photo-induced charge carriers. The PL spectra were also carried out to probe the recombination of photo-generated charge carriers (Li et al., 2020b; Nie et al., 2020). Compared with BOI and 6EBOI samples, the lowest intensity of 6Y6EBOI suggests that it possesses the lowest recombination rate of photo-generated charge carriers, which is beneficial to improve the photocatalytic activity (Figure 11B). According to above results, the Er^3+^ and Yb^3+^ doping into Bi_5_O_7_I samples shows enhancing photocatalytic degradation activities for BPA.

### Photocatalytic Mechanism

**Figure 12:**
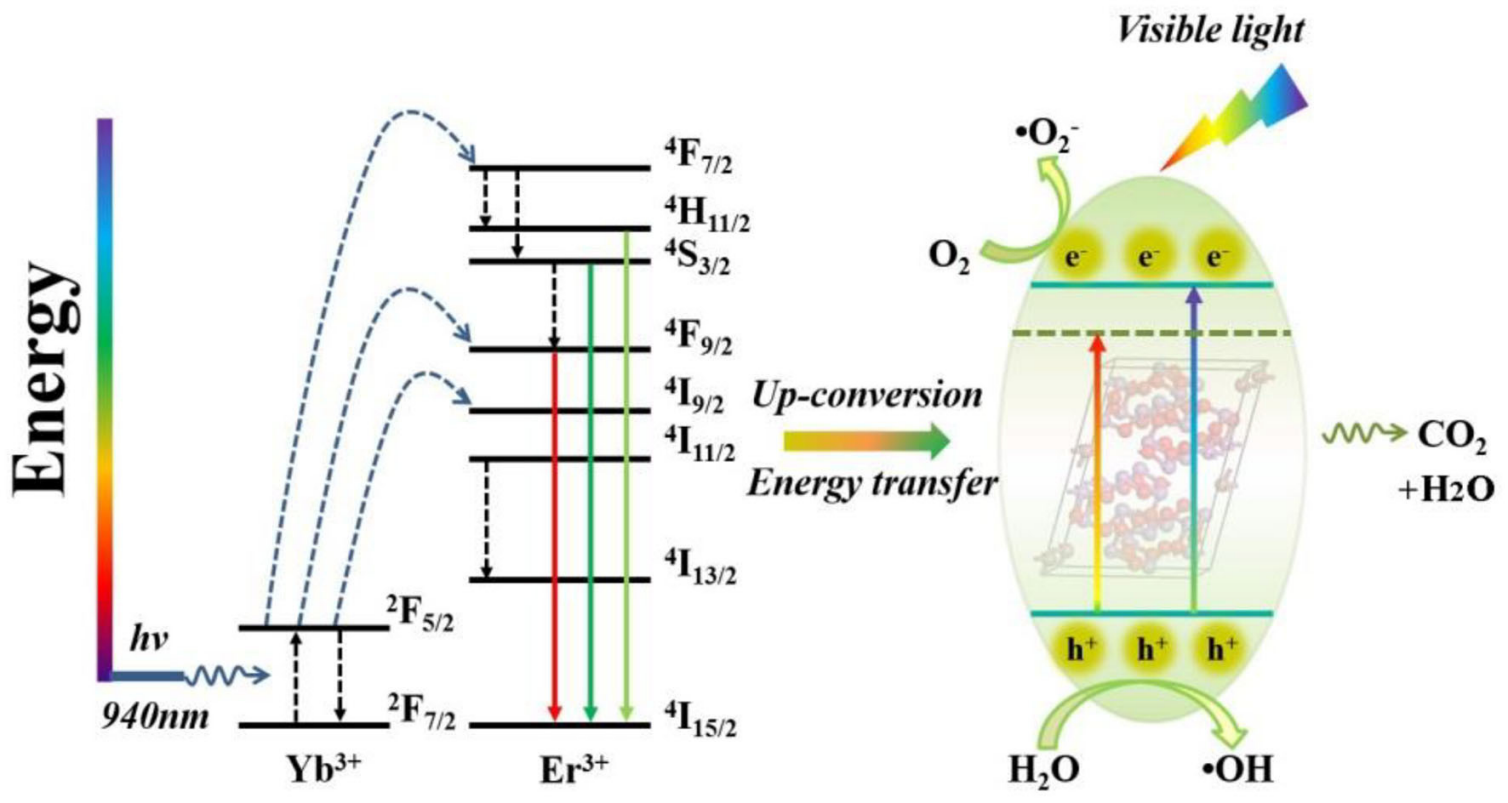
Schematic photodegradation mechanisms over 6Y6EBOI sample under visible light irradiation.

Figure 12 illustrates the photocatalytic reaction mechanism of the Yb^3+^/Er^3+^-doped Bi_5_O_7_I photocatalyst. It can be seen that the Yb^3+^/Er^3+^-doped Bi_5_O_7_I sample could absorb low-energy IR light, and then the electrons would be excited from the level of ^2^F_7/2_ to ^2^F_5/2_. Then, the excited electrons would be transferred back to the ground state of ^2^F_7/2_, and the energy released in this process is mainly transferred to the active Er^3+^ in a non-radiative manner, leading to a population of Er^3+^ from ^4^I_15/2_ to ^4^I_11/2_ (Wu et al., 2013). Next, a second or more similar photons from excited Yb^3+^ may convert to higher ^4^F_9/2_, ^4^F_7/2_, and ^2^I_9/2_ energetic levels of Er^3+^. Then, some of the excited electrons will relax non-radiatively to the energy levels of ^2^H_11/2_, ^4^S_3/2_, ^4^F_9/2_ etc. energy levels through a fast multiphonon decay process (Lei et al., 2015), leading to a stronger green (^2^H_11/2_, ^4^S_3/2_-^4^I_15/2_) and red emission (^4^F_9/2_-^4^I_15/2_), especially the latter. Therefore, the improvement in photocatalytic efficiency of the YEBOI samples could be elaborated more clearly in three factors. First, the Yb^3+^/Er^3+^doping in the photocatalyst can cause significant redshift with the absorption of visible light, which would excite more electron-hole pairs. Second, the upconversion process in Yb^3+^/Er^3+^-doped Bi_5_O_7_I sample will take place, and it will produce electron-hole pairs under low-energy IR light irradiation. In this process, the photoactivity of Yb^3+^/Er^3+^-doped Bi_5_O_7_I sample is evidently enhanced. Third, the Yb^3+^ and Er^3+^ in the Bi_5_O_7_I would promote the separation of e^−^/h^+^ pairs, so more photo-induced charge carriers would migrate to the sample surface for photocatalytic reaction (Zhang et al., 2012).

## Conclusions

In this work, the Yb^3+^/Er^3+^-doped Bi_5_O_7_I microsphere photocatalysts were prepared through combining hydrothermal and heat-treatment method. The Yb^3+^/Er^3+^-doped Bi_5_O_7_I photocatalysts have excellent photocatalytic for BPA under visible light irradiation and upconversion luminescence properties. It is expected that the synthetic method and properties of this catalyst will offer some inspiration and help for the future researchers to improve similar photocatalytic and upconversion luminescence materials.

## Data Availability Statement

The raw data supporting the conclusions of this article will be made available by the authors, without undue reservation.

## Author Contributions

GZ designed the project. BC, SG, and LL performed the experiments. BC, SG, and RD performed the data analysis. GZ, YX, and LG contributed to the theoretical analysis. BC, SG, and SZ wrote the paper. All authors contributed to the general discussion, contributed to the article, and approved the submitted version.

## Conflict of Interest

The authors declare that the research was conducted in the absence of any commercial or financial relationships that could be construed as a potential conflict of interest.
