# Supplementary material for: Fabrication of Er3+/Yb3+ Co-Doped Bi5O7I Microsphere With Upconversion Luminescence and Enhanced Photocatalytic Activity for Bisphenol A Degradation
Source: Front Chem. 2020 Sep 3;8:773. doi: 10.3389/fchem.2020.00773 (PMC7496766; doi:10.3389/fchem.2020.00773)
Supplement: Supplementary file 1 [file Data_Sheet_1.doc]

**Supporting Information**

**Fabrication of Er^3+^/Yb^3+^ co-doped Bi_5_O_7_I microsphere with** **up-conversion luminescence and** **enhanced photocatalytic activity for bisphenol A degradation**

Baowei Cao^1^, Siwen Gong^2^, Siyaka Mj Zubairu^3^, Lingna Liu^1^, Yunhua Xu^1^, Lei Guo^1^, Rui Dang^1^, Gangqiang Zhu^1,2^*

*^1^School of Chemistry and Chemical Engineering, YuLin University, Yulin, China*

*^2^School of Physics and Information Technology, Shaanxi Normal University, Xi’an 710062, PR China*

*^3^Department of Chemistry, Federal University Gashua PMB 1005, Gashua, Yobe State, Nigeria*

*Corresponding author: Tel/Fax: +86-29-81530750; Email address: zgq2006@snnu.edu.cn

1. **Experimental**
   1. *. Synthesis of Bi_5_O_7_I and Er^3+^ doped Bi_5_O_7_I*

All the chemical reagents were of analytical grade and used without further purification. The samples were first prepared by the ethylene glycol-assisted solvothermal and then calcinated at 450^o^C. In a typical synthetic procedure, amount of Er(NO_3_)_3_•6H_2_O, 1.3825 g of Bi(NO_3_)_3_•5H_2_O and 0.5010 g of KI were separately dissolved in 25 mL ethylene glycol under ultrasonication for 15 min. Then, the two solutions were mixed under vigorous stirring for 50 min, and transferred into a Teflon-lined stainless steel autoclave for reaction at 160^o^C for 12 h. The resulting precipitates were washed with deionized water and ethanol for several times, and dried at 75^o^C for 12 h. The obtained precipitates were heated at 450^o^C for 2 h in air and the as-prepared samples were denoted as BOI and 6EBOI, respectively.

- 1. *.* Characterization

The crystalline phases were determined using X-ray powder diffraction (XRD), using Cu K1 (k=1.5418Å) radiation at a scanning rate of 10° min-1 in the 2θ range of 10° to 70°. The UV-vis diffuse reflectance spectra were acquired using a UV-VIS-NIR spectroscopy (Cary 5000, Agilent) using BaSO_4_ as the background over a range of 200-1200nm. The photoluminescence spectra (PL) were obtained by a Fluoromax-4 spectrophotometer (HORIBA Scientific). The X-ray photoelectron spectroscopy (XPS) were obtained by ESCALAB MKII spectrometer (VG Scienta) with Al Ka radiation (hv =1486.6 eV).

- 1. *. Photocatalytic test*

The photocatalytic performance was evaluated by the degradation of bisphenol A (BPA) solution under light irradiation using a LED photo-reactor (PCX50A discover, Beijing Perfect Light Technology co., LTD). A 3W (λ=400-780 nm) and a 100W 940 nm LED lights were used as visible and NIR light sources, respectively. Besides, two different LED strips were also used in this work, namely: green light (λ=525 nm) and red light (R-LED, λ=630 nm). The decomposition of the pollutants was carried out with 50 mg of the catalyst dispersing uniformly into 50 mL bisphenol A (BPA=15 mg/L) in a quartz catalyst tube, respectively. Prior to irradiation, the as-prepared samples as photocatalysts for BPA were continuously stirred in the dark for 30 min to ensure an adsorption-desorption equilibrium. During the irradiation, 2 mL of suspension was taken out at a given interval for subsequent pollutant concentration analysis by using a U-3010 UV-vis spectrophotometer. The wavelength of BPA was detection at 277 nm**.**

1. **Results**


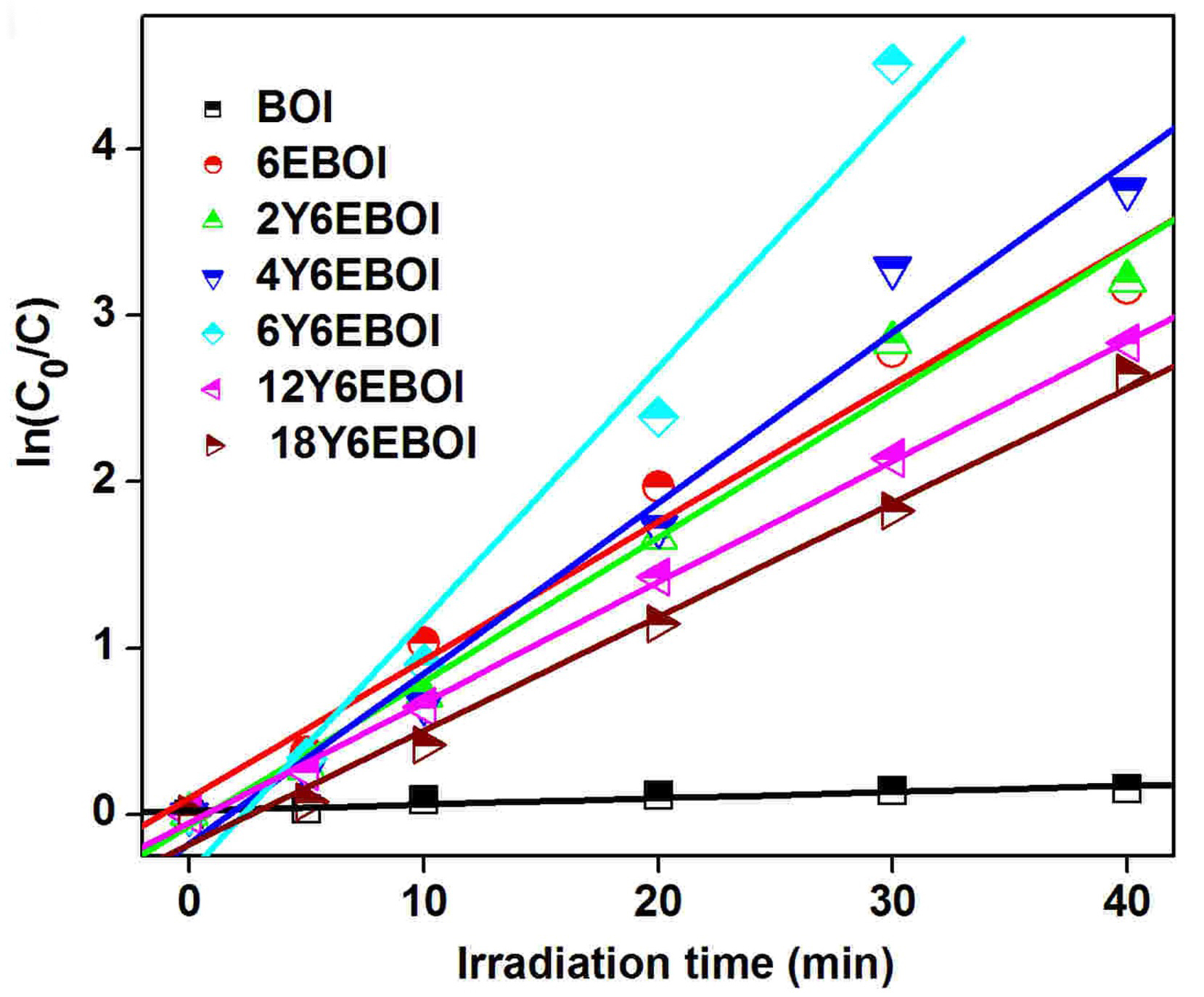


**Figure S1.** The kinetics study for BOI, 6EBOI, 2Y6EBOI, 4Y6EBOI, 6Y6EBOI, 12Y6EBOI, and 18Y6EBOI samples under visible light irradiation


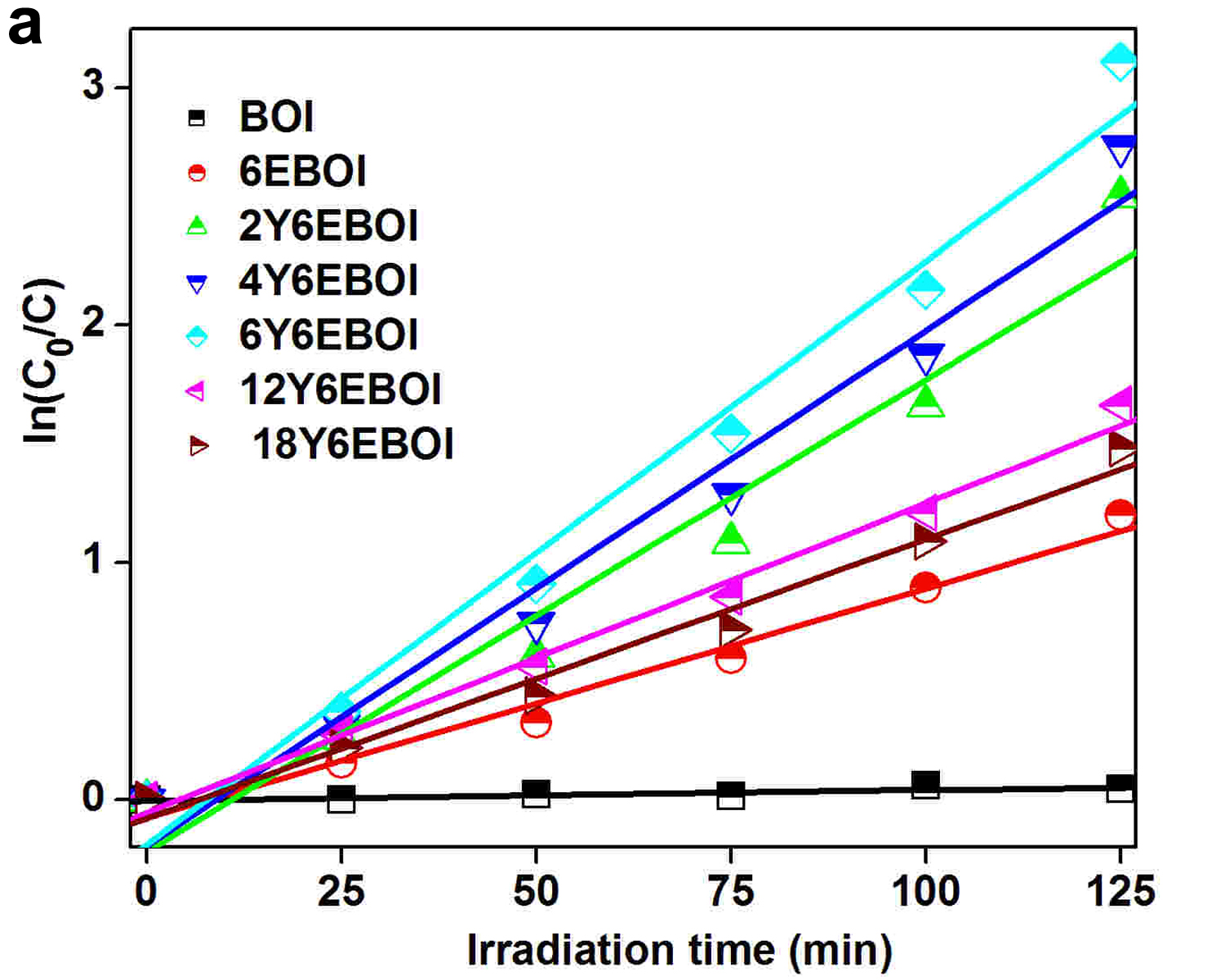

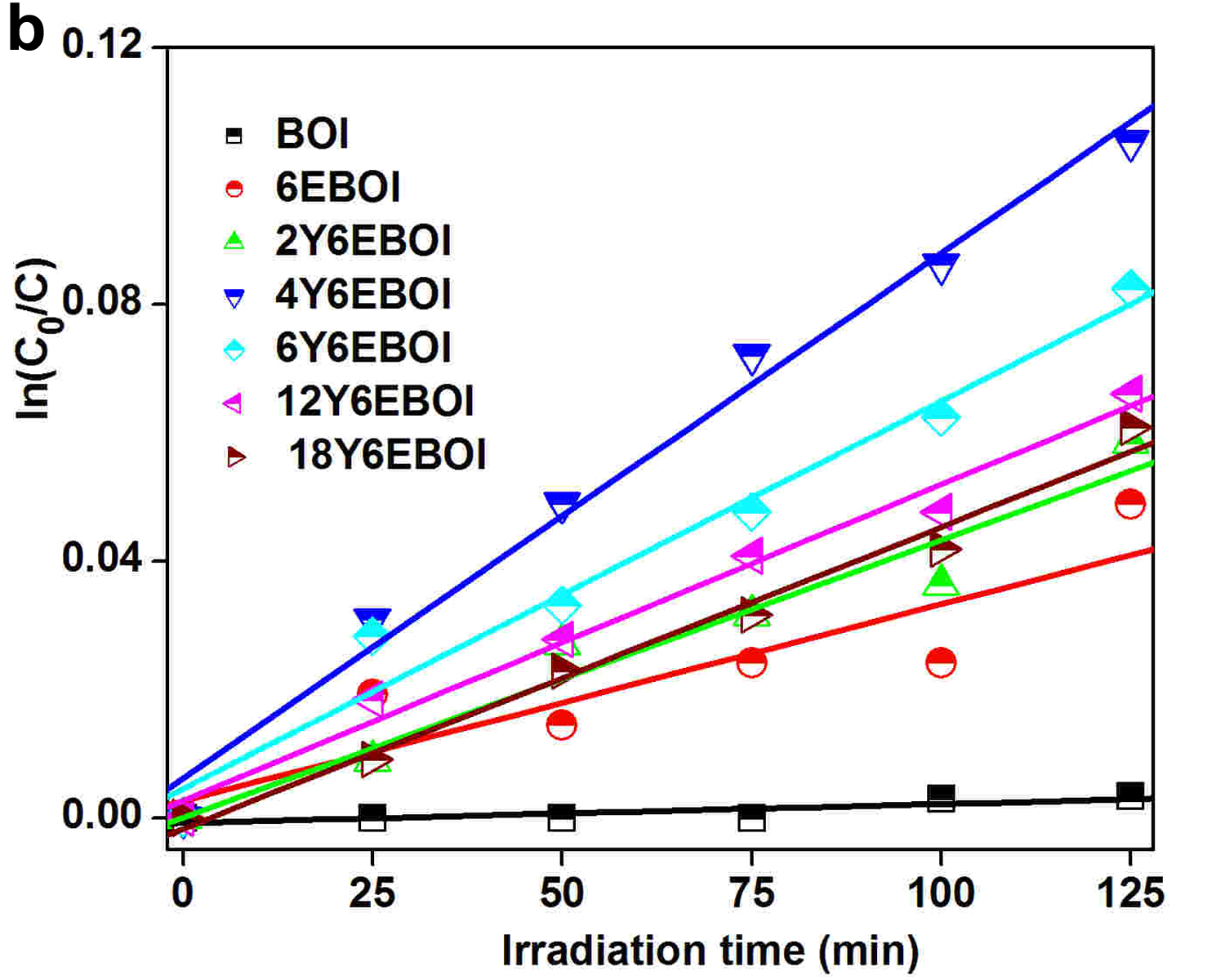


**Figure S2.** The kinetics study for BOI, 6EBOI, 2Y6EBOI, 4Y6EBOI, 6Y6EBOI, 12Y6EBOI, and 18Y6EBOI samples under green (a) and red (b) light irradiation
